# Navigating the complexity of Guillain-Barré syndrome and Miller-Fisher syndrome overlap syndrome: a pediatric case report

**DOI:** 10.11604/pamj.2024.47.127.42985

**Published:** 2024-03-22

**Authors:** Kamal Chafiq, Youssef Hadzine, Adel Elmekkaoui, Othmane Benlenda, Houssam Rajad, Soukaina Wakrim, Hicham Nassik

**Affiliations:** 1 Department of Anesthesiology and Reanimation, Faculty of Medicine and Pharmacy of Agadir, University Ibn Zohr, Agadir, Morocco,; 2 Department of Radiology, Faculty of Medicine and Pharmacy of Agadir, University Ibn Zohr, Agadir, Morocco

**Keywords:** Guillain-Barré syndrome, Miller-Fisher syndrome, overlap syndrome, case report

## Abstract

Guillain-Barré syndrome/Miller-Fisher syndrome (GBS/MFS) overlap syndrome is an extremely rare variant of Guillain-Barré syndrome (GBS) in which Miller-Fisher syndrome (MFS) coexists with other characteristics of GBS, such as limb weakness, paresthesia, and facial paralysis. We report the clinical case of a 12-year-old patient, with no pathological history, who acutely presents with ophthalmoplegia, areflexia, facial diplegia, and swallowing and phonation disorders, followed by progressive, descending, and symmetrical paresis affecting first the upper limbs and then the lower limbs. An albuminocytological dissociation was found in the cerebrospinal fluid study. Magnetic resonance imaging of the spinal cord showed enhancement and thickening of the cauda equina roots. The patient was treated with immunoglobulins with a favorable clinical outcome.

## Introduction

Guillain-Barré syndrome (GBS) is an acute polyradiculoneuropathy, classically ascending with an autoimmune mechanism. Guillain-Barré syndrome can manifest in several subtypes and clinical variants, each presenting a distinct set of clinical features. Miller-Fisher syndrome (MFS) represents a very rare regional variant of GBS, characterized by the classical triad: ophthalmoplegia, ataxia, and areflexia, with an incidence of 1 to 2 cases per million inhabitants [1]. Miller-Fisher syndrome can coexist with other features of GBS, such as limb weakness, paresthesia, and facial paralysis, thus constituting an even rarer variant, known as GBS/MFS overlap syndrome [1,2]. We report a pediatric clinical case of a patient presenting with GBS/MFS overlap following a respiratory infection.

## Patient and observation

**Patient information:** we present a case of a 12-year-old child, born of non-consanguineous parents, immunocompetent with good psychomotor development and without notable medical history until the day of hospitalization in the intensive care unit due to respiratory distress following the sudden onset of neurological symptoms including horizontal diplopia, phonation difficulties, swallowing issues, facial hypomimia, and progressive, symmetric, descending muscle weakness affecting both upper and lower limbs, leading to an inability to walk within 3 days. Two weeks before admission, the patient reported fever and respiratory symptoms such as cough with whitish sputum, associated with diffuse abdominal pain without vomiting or bowel disturbances.

**Clinical findings:** the clinical examination revealed fever, tachypnea, respiratory distress with oxygen saturation of 90% on room air, and periumbilical abdominal tenderness. Neurological examination revealed a global neurological deficit, more pronounced proximally, with reduced muscle strength rated 3/5 in the upper limbs and 4/5 in the lower limbs, absent osteotendinous reflexes, facial diplegia, and bilateral convergent strabismus with the absence of cough and swallowing reflexes.

**Figure 1:**
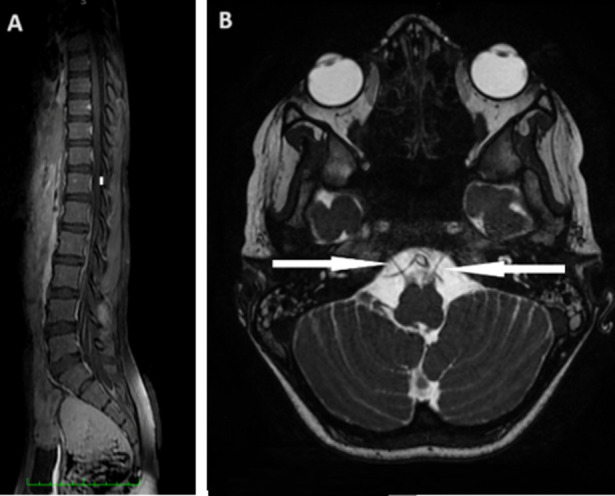
the sagittal T1-weighted MRI with gadolinium enhancement; A) showing enhancement and thickening of the cauda equina roots; the axial FIESTA MRI with gadolinium enhancement; B) demonstrating a right-angled crossing with direct contact between the anterior inferior cerebellar artery and the abducens nerve (VI) bilaterally

**Diagnostic assessment:** on a biological level, there is evidence of leukocytosis with elevated C-reactive protein and procalcitonin levels. Tuberculosis screening was negative. Cytobacteriological examination of sputum revealed sterility. Serologies for HIV, cytomegalovirus, and Epstein-Barr virus (EBV) were negative. Immunoglobulin levels (IgA, IgM, and IgG) were normal. Lymphocyte subpopulation levels (CD3, CD4, and CD8) were low. Anti-DNA antibodies and antinuclear antibodies were normal. Lumbar puncture showed elevated protein levels without cellular abnormalities, suggestive of albuminocytological dissociation. Anti-GQ1b antibody testing was positive. The brain and spinal cord magnetic resonance imaging showed enhancement with thickening of the cauda equina roots and a right-angled crossing with direct contact between the anterior inferior cerebellar artery and the abducens nerve (VI) bilaterally (Figure 1).

**Diagnosis:** we considered the diagnosis of GBS/MFS overlap syndrome due to the acute onset of ophthalmoplegia, areflexia, facial diplegia, and swallowing difficulties followed by descending and symmetric muscle weakness involving all four limbs, along with the presence of albuminocytological dissociation in the cerebrospinal fluid and positivity for anti-GQ1b antibodies, as well as enhancement and thickening of the cauda equina roots on spinal magnetic resonance imaging.

**Therapeutic interventions:** given the clinical presentation and progression of symptoms, the patient received a 5-day course of intravenous immunoglobulin (15 grams per day) as treatment. Additionally, supportive care was provided, including topical lubricating eye drops, and eye patches and a nasogastric tube was placed for feeding purposes. The patient underwent 4 weeks of inpatient rehabilitation to address persistent gait difficulties and limb weakness.

**Follow-up and outcome of interventions:** the clinical progression was favorable under treatment. By the fourth day, there was a regression of swallowing difficulties, facial diplegia, and diplopia. Within a week, the patient began to walk with assistance.

**Patient perspective:** the family was initially engulfed in fear of the unknown and the rapid decline of their daughter's health. The treatment, intravenous immunoglobulins, known for its efficacy, was unfortunately also known for its high cost-a cost prohibitive for the family, causing an additional layer of distress. However, in the sanctuary of the hospital, they were provided with this essential medication. The family, already bracing for the worst, found solace in the hospital's capacity to administer the treatment. Their relief was palpable as they saw their daughter's condition improve significantly. This turn of events, facilitated by the hospital, brought clinical recovery and a deeply appreciated emotional respite from the financial strain they faced outside the healthcare facility.

**Informed consent:** it was obtained from the patient´s family to publish this work.

## Discussion

Guillain-Barré syndrome (GBS) is an acute immune-mediated peripheral neuropathy, the leading cause of acute flaccid paralysis, with a global incidence of 1 to 2 cases per 100,000 people per year. Guillain-Barré syndrome is classically characterized by rapidly progressive, symmetric, ascending muscle weakness with areflexia and albuminocytological dissociation in the cerebrospinal fluid [2].

In addition to the classical form of GBS, several variants exist. These include regional variants known as regional variants, which are: the pharyngo-cervical-brachial form, bilateral facial weakness with limb paresthesia, and the para-paretic variant. The spectrum of MFS can present either as the typical form of MFS, which is a rare descending paralysis characterized by the triad: ophthalmoplegia, areflexia, and ataxia, or as incomplete forms of MFS (without all three criteria of the triad) [2,3]. Currently, there is a discussion of the GBS/MFS overlap syndrome, which is itself a variant of GBS that combines MFS with limb weakness [2]. The clinical case we are reporting presents clinical symptoms consistent with GBS/MFS overlap syndrome.

Guillain-Barré syndrome is less common in children and adolescents than in the adult population. In this population, the overall incidence is estimated at 0.62 cases per 100,000 individuals aged 0 to 9 years, and 0.75 cases per 100,000 individuals in the age group of 10 to 19 years, with a clear male predominance [4]. Miller-Fisher syndrome represents a very rare form, with an overall incidence of 1 to 2 cases per million individuals, also showing a male predominance [1]. An East Asian study has shown that the incidence of Miller-Fisher syndrome is higher in East Asia, especially in the pediatric population, where it accounts for up to 25.4% of children with GBS, with an incidence of 44% for GBS/MFS overlap syndrome [5].

Guillain-Barré syndrome (GBS) is considered a post-infectious autoimmune disease, most commonly preceded by a respiratory or gastrointestinal infection. The most commonly described infectious agents include campylobacter jejuni, cytomegalovirus, *Mycoplasma pneumoniae*, Epstein-Barr virus, *Haemophilus influenzae*, and enteroviruses have also been linked to GBS [1]. More than 90% of patients with MFS have anti-GQ1b IgG antibodies during the acute phase [6]. A GQ1b is a specific type of ganglioside, a tetrasialoganglioside found on the cell surface of the central and peripheral nervous system but highly enriched at the nodes of Ranvier of cranial nerves III, IV, and VI [7]. The damage caused by these antibodies to the peripheral nerves correlates well with the anti-GQ1b antibody titer [8].

The diagnosis of GBS/MFS overlap syndrome was clinically established due to the acute onset of ophthalmoplegia, areflexia, facial diplegia, and swallowing difficulties followed by progressive, descending, and symmetric paresis affecting all four limbs. This clinical presentation was reinforced by the presence of albuminocytological dissociation in the cerebrospinal fluid and positivity for anti-GQ1b antibodies. As in the case of our patient, involvement of other cranial nerves, particularly bilateral facial nerve involvement, can occur [2,5]. Spinal magnetic resonance imaging (MRI) is not routinely used but may be employed in certain situations to exclude differential diagnoses or, as in our case, to support the diagnosis by showing enhancement of the spinal nerve roots or cauda equina [1].

The treatment relies on appropriate supportive care, including pain control and respiratory support. Intravenous immunoglobulins (IVIg) and plasmapheresis (PE) are effective treatments for GBS. No study has demonstrated a significant difference in terms of efficacy between these two treatments, but it is strongly recommended to start with immunoglobulins in patients unable to walk or with swallowing difficulties [2].

The prognosis is generally favorable with good clinical improvement [1,2]. A short interval of time between symptom onset and hospitalization predicts a less favorable outcome. Hospitalization in an intensive care unit or the need for intubation is not indicative of a higher risk of sequelae [9]. Residual symptoms may persist long-term in approximately 67% of patients, with the most common being fatigue, pain, and paresthesia [10].

## Conclusion

The MFS in children is rare and should be considered in the presence of the classic triad of ophthalmoplegia, ataxia, and areflexia. Incomplete forms can complicate the diagnosis, especially when associated with other symptoms of GBS, thus resulting in an overlap syndrome. This clinical case highlights the importance of measuring anti-GQ1b antibodies in diagnosing MFS and, consequently, GBS/MFS overlap syndrome. Early recognition and appropriate treatment are essential to improve prognosis and reduce sequelae.
